# RAS/BRAF Circulating Tumor DNA Mutations as a Predictor of Response to First-Line Chemotherapy in Metastatic Colorectal Cancer Patients

**DOI:** 10.1155/2018/4248971

**Published:** 2018-03-07

**Authors:** Jiannan Yao, Wanchun Zang, Yang Ge, Nathaniel Weygant, Pan Yu, Lei Li, Guanhua Rao, Zhi Jiang, Rui Yan, Linjia He, Yang Yu, Mulan Jin, Gang Cheng, Guangyu An

**Affiliations:** ^1^Department of Oncology, Beijing Chao-Yang Hospital, Capital Medical University, Beijing, China; ^2^Beijing Novogene Bioinformatics Technology Co., Ltd., Beijing, China; ^3^Department of Medicine, The University of Oklahoma Health Sciences Center, Oklahoma City, OK, USA; ^4^Department of Pathology, Beijing Chao-Yang Hospital, Capital Medical University, Beijing, China

## Abstract

**Background:**

Since circulating tumor DNA (ctDNA) offers clear advantages as a minimally invasive method for tumor monitoring compared with tumor tissue, we aimed to evaluate genotyping ctDNA using a next-generation sequencing- (NGS-) based panel to identify the prognostic value of mutation status in metastatic colorectal cancer (mCRC) patients with primary tumor resected and with subsequent lines of treatment in this study.

**Methods:**

76 mCRC patients treated in Beijing Chao-Yang Hospital from 2011 to 2017 were enrolled. Genotyping of RAS/BRAF in tumor tissue and ctDNA was determined by ARMS PCR and with a 40-gene panel using NGS, respectively. Patient clinicopathologic features and RAS/BRAF gene mutation status were evaluated by survival analysis for disease-free survival (DFS) and progression-free survival (PFS).

**Results:**

Among 76 patients, KRAS distributions were not significantly correlated with any clinicopathologic features. The concordance between tumor tissue and ctDNA KRAS mutation was 81.25%. Mutations of RAS/BRAF had no significant impact on DFS after surgery (hazard ratio (HR), 1.205; 95% CI, 0.618 to 2.349; *P* = 0.5837) but prognosticated poorer PFS in subsequent first-line therapy (HR, 3.351; 95% CI, 1.172 to 9.576; *P* = 0.024).

**Conclusion:**

ctDNA was comparable with tumor tissue for mutation detection. RAS/BRAF mutations detected in ctDNA predict a worse PFS in mCRC patients with first-line chemotherapy. Our results provide support for the prognostic value of RAS/BRAF ctDNA mutation detection in mCRC patients.

## 1. Introduction

Colorectal cancer (CRC) is one of the most common malignancies worldwide [1, 2]. In the past decade, the 5-year survival rate for CRC patients has improved mainly due to the development of new drug combinations and targeted molecular agents. Several clinical trials have proven that EGFR monoclonal antibody (mAb) improves survival in KRAS wild type CRC patients relative to standard chemotherapy, while patients with RAS-mutant tumors derive no benefit [2–5]. Current clinical guidelines recommend that all metastatic CRC (mCRC) patients being considered for EGFR mAb therapy should have the confirmed absence of relevant RAS/BRAF mutations [6].

Although testing for RAS/BRAF mutation is recommended before targeted therapy selection, the prognostic value of these mutations remains unclear. In patients with stage II and III CRC who underwent curative resection followed by standard adjuvant chemotherapy (FOLFOX or FOLFIRI), KRAS mutation was significantly associated with poor disease-free survival (DFS), while the BRAF mutation was not associated with DFS [7]. Moreover, results of the N0147 trial suggest that KRAS mutations in either codon 12 or codon 13 are associated with reduced DFS in resected stage III colon cancer patients [8]. However, another study reported that KRAS mutation status was not a significant factor in the progression-free survival (PFS) and overall survival (OS) of patients with synchronous mCRC [9].

Primary tumor tissue is used for RAS/BRAF genotyping, which is widely accepted as a basis for the treatment of mCRC because of high concordance (93–97%) between primary tumor and metastases [10, 11]. However, a noninvasive, dynamic and reliable genotyping method would be beneficial if mutational status could be confirmed consecutively, especially in cases of progressing disease patients or those with complete resection of primary tumor tissue. CtDNA makes minimally invasive, dynamic genotyping and consequently prognostic predictions possible, and next-generation sequencing (NGS) enables rapid and highly sensitive identification of somatic genomic alterations in individuals. Therefore, we designed this analysis to validate the use of an NGS-based panel to identify the correlation between targeted genotyping of ctDNA and tissue-derived DNA at different time points. In addition, we aimed to explore the prognostic value of RAS/BRAF gene status in CRC patients with primary tumor resected and with subsequent lines of chemotherapy.

## 2. Materials and Methods

### 2.1. Patients

A total of 76 patients diagnosed with CRC from March 1, 2013, to Nov 17, 2016, at Beijing Chao-Yang Hospital were included in this study. There were 48 patients who received standard combination chemotherapy (oxaliplatin or irinotecan based), and patients receiving EGFR mAb or other biological treatments were excluded. Patient characteristics were retrospectively collected from hospital records. Pathological characteristics of patient tumors including histologic grade, nodal invasion, vascular tumor thrombus, and perineural invasion were also collected. The TNM and NCCN stage was categorized according to the AJCC Staging Manual and NCCN guidelines, respectively. Response of treatment was evaluated by the Response Evaluation Criteria in Solid Tumors (RECIST). The last day of follow-up was January 1, 2017. Patients without documented progression were censored at the last tumor assessment or follow-up. This study was approved by the human research ethnical board from Beijing Chao-Yang Hospital and written informed consent was obtained from all the patients.

### 2.2. Sample Collection

Tumor tissue samples were collected from all patients prior to therapy during surgery or biopsy. Patients were classified into two groups according to different plasma and tissue collection time points: synchronous group (plasma samples collected within 7 days of tumor tissue) and metachronous group (plasma samples collected within the course of subsequent lines of treatment).

Pretreatment tumor biopsies and resected tumor tissues were fixed in formalin and preserved in paraffin blocks for histological examination and DNA extraction. Collected formalin-fixed, paraffin-embedded (FFPE) specimens were reviewed by a pathologist. Microdissection was carried out to confirm the sufficiency of tumor cells when specimens contain less than 50% tumor area. Genomic DNA was extracted from FFPE samples using Biomark DNA FFPE Kit (Beijing ACCB Biotech Ltd., China) according to the manufacturer's protocol.

**Figure 1:**
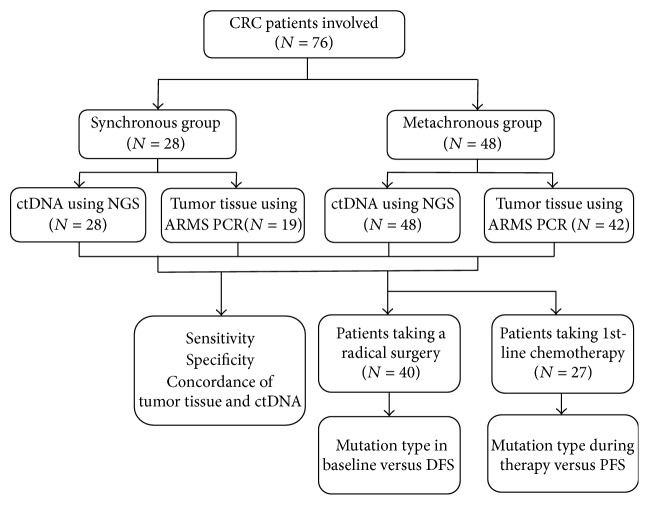
Overview of the work flow.

Blood samples were collected in 10 ml BD Vacutainer plastic tubes containing EDTA. The plasma fraction was separated from the blood cells within 2 h after blood draw by centrifugation at 1900 g for 10 min at 4°C. Plasma samples were transferred to a new tube and centrifuged at 16000 g for 10 min at 4°C to remove cellular debris. Plasma ctDNA was extracted using the QIAamp Circulating Nucleic Acid Kit (Qiagen, Valencia, CA). The purity and concentration of extracted DNA were determined by spectrophotometry. The DNA samples with absorption ratios of 260/280 nm greater than 1.8 were used for subsequent analyses. DNA was stored in −80°C for future use. The workflow is summarized in Figure 1.

### 2.3. Tumor DNA Mutation Analysis by Amplification Refractory Mutation System-Polymerase Chain Reaction (ARMS PCR)

The ARMS PCR assay was performed to detect mutations in codons 12, 13, 59, 61, 117, and 146 of KRAS and NRAS and codon 600 of BRAF using the Human KRAS/NRAS/BRAF Mutations Detection Kit (Beijing ACCB Biotech Ltd., China) according to manufacturer's manual in the pathology department. The analytical sensitivity of this assay is 1%.

### 2.4. NGS and Data Processing

30–50 ng ctDNA was used to amplify a targeted ctDNA library produced by Agilent's SureSelect QXT (Agilent Technologies, Santa Clara, CA, USA) according to the manufacturer's instructions. The captured DNA was sequenced using Illumina Hiseq 2500 platform (Illumina, CA, USA) with paired-end reads of 150 bp according to the manufacturer's instructions. The cleaned reads were mapped to the human reference genome (hg19) using BWA-mem software (version 0.7.8). Duplicate PCR reads were marked using Picard MarkDuplicates (https://picard.sourceforge.net/). Variant calling was performed using samtools (http://samtools.sourceforge.net/), and variants were annotated using ANNOVAR (http://annovar.openbioinformatics.org/en/latest/). The sequencing quality controls were as follows:* Q*20 > 90%;* Q*30 > 85%; coverage of target region > 99%; mapping rate ≥ 95%; and average coverage per sample higher than 2000x. We analyzed nonsynonymous somatic mutations in KRAS/NRAS/HRAS/BRAF. Somatic mutations with a frequency lower than 5% were manually reviewed using the Integrative Genomics Viewer (IGV) software (http://software.broadinstitute.org/software/igv/), which were deemed positive when there were an abundance higher than 0.5% and more than 4 mutated reads from the paired group.

### 2.5. Statistical Analysis

The Mann–Whitney test and chi-square test were used for numerical and categorical variable comparison, respectively. Concordance of KRAS mutation status between the primary tumor and corresponding plasma ctDNA was analyzed by calculating the proportion of total true positive and true negative results out of all samples tested. The Kaplan-Meier methods were used to estimate survival curve. Associations of mutation status or other clinicopathologic features with DFS and PFS was analyzed by applying the maximum likelihood estimation (MLE). DFS was defined as the length of time after radical surgery treatment that the patient survives without any signs or symptoms of tumor recurrence. PFS was defined from the date of plasma collection to objective tumor progression or censoring at last follow-up. All statistical tests were two-sided and *P* values below 0.05 were considered to be statistically significant. Statistical analyses were performed using SAS version 9.2 (SAS Institute, Cary NC, USA).

## 3. Results

### 3.1. Patient Characteristics

A total of 76 CRC patients were included in this study. The median patient age was 62 years, ranging from 25 to 82 years. The majority gender enrollment was male with 64.47% (49/76). The location of the primary tumors was mostly rectum and sigmoid colon with 65.79% (50/76). 84.00% (63/76) of patients accepted surgical resection of the primary tumor. The tumors were graded as follows: 5.26% (4/76) were well differentiated, 63.16% (48/76) were moderately differentiated, 11.84% (9/76) were poorly differentiated, and 19.74% (15/76) were not available. Patient characteristics are summarized in Table 1.

### 3.2. RAS and BRAF Mutation Detected in Tissue and ctDNA

The gene mutations in both primary colorectal tumor tissue DNA and ctDNA were analyzed. Due to low amounts of DNA from FFPE samples, mutational data were available in 61 of 76 tumor tissue samples. All patients had plasma ctDNA detection results. With respect to tumor tissue, the KRAS mutation rate was 40.98% (25/61), while it was 32.90% (25/76) in ctDNA. Comparison of KRAS mutation distribution between patient subgroups was performed. KRAS mutations in tissue or in ctDNA had no association with clinicopathologic characteristics (Table 1). The most frequently detected KRAS mutations were in codons 12 and 13 in both tumor tissue and ctDNA. Among 76 patients, we detected BRAF mutations Y472fs, P403fs in 2 (2.63%) patients, HRAS in 3 (3.95%) patients, and NRAS in 2 (2.63%) patients. Mutations detected are summarized in Table 2.

KRAS was the most commonly mutated gene to be identified in both tumor tissue and ctDNA, and we used KRAS to evaluate the concordance of ctDNA with tumor tissue. There were 19 of 28 patients in the synchronous group that had tumor tissue mutations detected using ARMS PCR. According to the KRAS detection, the ctDNA had a sensitivity of 66.67% (4/6), specificity of 87.50% (14/16), and concordance of 81.82% (18/20) compared with tumor tissue. In 42 of 48 patients in the metachronous group who had ARMS PCR results, the ctDNA had a sensitivity of 66.67% (12/18), specificity of 91.67% (22/24), and concordance of 80.95% (34/42) compared with tumor tissue. In total, there were 8 patients with KRAS mutation in tumor tissue that was not detected in plasma and 4 patients with plasma KRAS mutations only. The sensitivity and concordance of ctDNA in synchronous and metachronous groups were identical. The overall sensitivity, specificity, and concordance were 66.67%, 90.00%, and 81.25%, respectively (Table 3).

### 3.3. Gene Mutation Status as a Prognostic for Therapy Treatment Response

**Figure 2:**
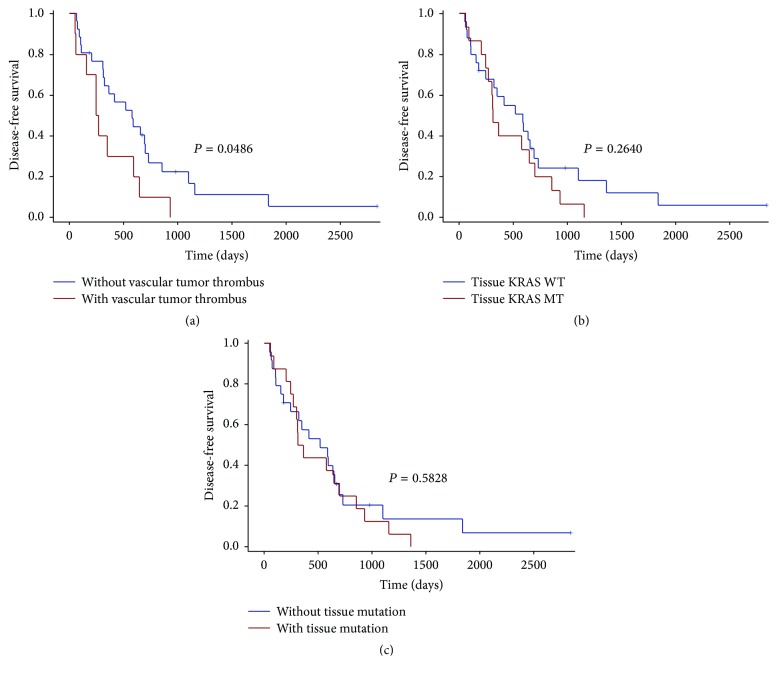
Kaplan-Meier plots of patients for disease-free survival (DFS) grouped by vascular tumor thrombus, KRAS mutation status, and RAS/BRAF mutation status. The DFS was significantly worse in patients with vascular tumor thrombus, with a median DFS of 259 days versus 578 days for nonvascular tumor thrombus patients (*P* = 0.0486) (a). There was no significant difference in DFS between KRAS mutant (MT) and wild type (WT) patients (median DFS 313 days versus 590 days, *P* = 0.2640) (b) or between RAS/BRAF mutant patients and wild type patients (median DFS 339 days versus 519 days, *P* = 0.5828) (c).

**Figure 3:**
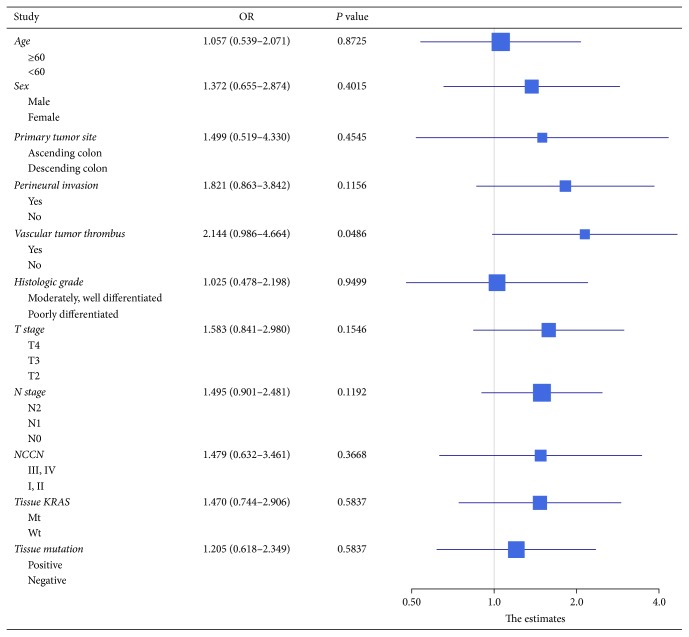
Forrest plot of hazard ratios for DFS of the surgical resection patients. Except for vascular tumor thrombus, none of the other clinicopathologic features showed significant association with poorer DFS.

**Figure 4:**
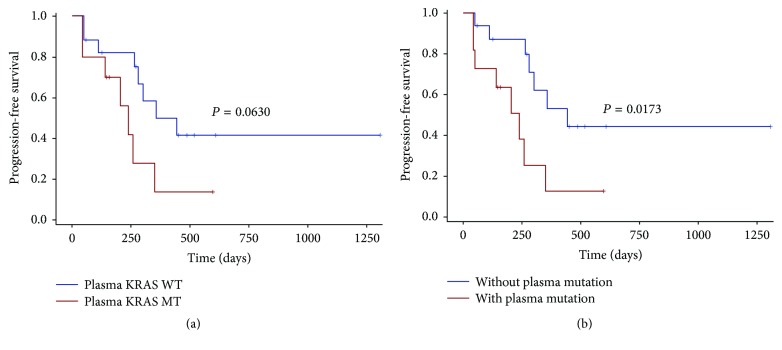
Kaplan-Meier plots of patients for progression-free survival (PFS) grouped by plasma KRAS and RAS/BRAF mutation status. The plasma KRAS mutations were not associated with shorter PFS (median PFS 239 days versus 443 days, *P* = 0.0630) (a). However, plasma RAS/BRAF mutations were associated with poor PFS compared to wild type (median PFS 239 days versus 443 days, *P* = 0.0173, HR = 3.351) (b).

**Figure 5:**
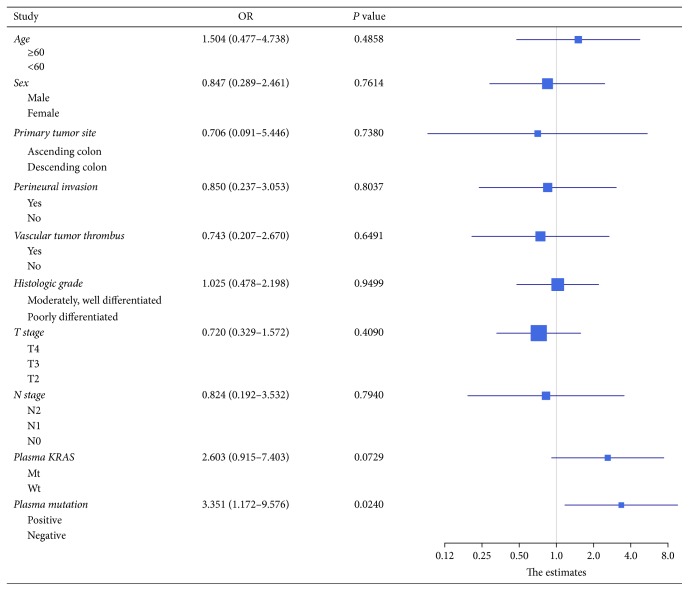
Forrest plot of hazard ratios for PFS of the subsequent first-line chemotherapy patients. Except for plasma RAS/BRAF mutations, other clinical factors including age, gender, primary tumor site, perineural invasion, vascular tumor thrombus, histologic grade, T stage, and N stage were not significantly associated with PFS.

We performed survival analysis to explore any factors that might influence the DFS and PFS of the CRC patients. 40 patients who underwent a radical surgery had measurable DFS (Supplement Table 1). The DFS was significantly lower in patients with vascular tumor thrombus, with a median DFS of 259 days versus 578 days for nonvascular tumor thrombus patients (*P* = 0.0486) (Figure 2(a)). There was no statistically significant difference in DFS between KRAS mutant and wild type patients (median DFS 313 days versus 590 days, *P* = 0.2640; Figure 2(b)) or between KRAS/NRAS/BRAF mutant patients and wild type patients (median DFS 339 days versus 519 days, *P* = 0.5828; Figure 2(c)). None of the other clinicopathologic features were significantly associated with DFS (Figure 3). We next assessed factors associated with PFS in subsequent first-line chemotherapy (Supplement Table 2). The survival analysis in 27 patients showed that plasma KRAS mutation was weakly associated with shorter PFS (median PFS 239 days versus 443 days, *P* = 0.0630; Figure 4(a)) but plasma RAS/BRAF mutations were significantly associated with shorter PFS compared to wild type RAS/BRAF (median PFS 239 days versus 443 days, *P* = 0.0173, HR = 3.351; Figure 4(b)). Other clinical factors including age, gender, primary tumor site, perineural invasion, vascular tumor thrombus, histologic grade, T stage, and N stage were not significantly associated with PFS (Figure 5).

## 4. Discussion

The goal of our study was to explore the prognostic value of RAS/BRAF gene status in blood ctDNA from CRC patients who are with resected primary tumors and treated with subsequent lines of palliative chemotherapy. Firstly, we validated the use of a NGS-based panel to identify mutations in ctDNA and explored the correlation of targeted genotyping in ctDNA and tissue-derived DNA at different time points. The results showed that ctDNA provides a surrogate to tumor tissue in genotyping. We further explored factors that might influence DFS and PFS, and we found that patients with RAS/BRAF ctDNA mutations in subsequent first-line therapy had poorer PFS than those without RAS/BRAF mutations. Prognostic and tumor monitoring applications may provide a rationale for the detection of RAS/BRAF ctDNA mutations in clinical practice.

In our study, KRAS was the most commonly mutated gene to be identified in both tumor tissue and ctDNA and accounted for 40.98% and 32.90% of mutations in tumor tissue and ctDNA, respectively. Besides KRAS mutation, other common mutations we detected have comparable frequencies reported in My Cancer Genome (https://www.mycancergenome.org/), which indicates that blood-based genomic profiling can effectively detect common mutations in CRC. As KRAS was the most commonly detected mutation, we compared the concordance of ctDNA and tumor tissue in detecting KRAS mutation in metachronous and synchronous groups. In both groups, the sensitivity of ctDNA was the same, and the concordance was also identical. The overall sensitivity, specificity, and concordance of ctDNA compared with primary tumor tissue DNA were 66.67%, 90.00%, and 81.25%. The sensitivity of ctDNA in the synchronous group (65.1%) was comparable to similar large sample size studies including those using ARMS PCR for ctDNA mutation detection [12]. The identical sensitivity in the metachronous group indicated that KRAS mutation was stable in primary tumor and blood ctDNA after disease recurrence, which may have applications in disease surveillance or therapy response evaluation. Clinical guidelines require that all mCRC patients being considered for EGFR mAb therapy should have confirmed absence of relevant KRAS/NRAS mutations. However, fresh biopsies require invasive techniques and are sometimes unavailable due to anatomic reasons, while multiple biopsies are often impractical when the original archived paraffin-embedded tissue samples are unavailable. Given these challenges, ctDNA genotyping may provide reliable information to improve clinical strategies.

It was previously reported that, in patients who underwent complete surgical resection, postoperative detection of ctDNA significantly correlated with RFS (*P* = 0.002, HR 3.1; 95% CI 1.7–9.1) and recurrence was detected in ctDNA a median of 5.1 months before radiographic recurrence [13]. In our study, we explored factors that might relate to recurrence after primary tumor resection. Firstly, we used KRAS mutation in tumor tissue as a baseline to evaluate its relationship with recurrence after primary tumor surgical resection. In our patient cohort, there was no significant correlation between KRAS mutation and recurrence. Aside from KRAS mutations, BRAF mutations have been shown to be markers of poor prognosis following mCRC treatment and have a more significant prognostic value than RAS mutations [14, 15]. As BRAF mutation cases are very rare in CRC patients [16, 17] and only two patients in this study had BRAF mutated, we could not analyze differences in DFS according to BRAF mutation. KRAS and BRAF are all downstream effectors of the EGFR [14], and tumors harboring RAS and BRAF mutations are unlikely to benefit from EGFR mAb therapy in mCRC [14, 18]. Thus, we took RAS and BRAF mutations together to assess its predictive value. It showed no statistically significant relationship between the RAS/BRAF mutations and recurrence from surgical resection, but we observed that patients with vascular tumor thrombus had worse DFS than patients without vascular tumor thrombus which was consistent with previous findings [19–21]. Since ctDNA KRAS had a concordance of 81.25% compared with tumor tissue in our study, we further aimed to analyze the prognostic role of ctDNA gene mutation status in subsequent first-line palliative chemotherapy. Patients with KRAS mutant ctDNA showed weakly statistical difference compared to those patients with wild type KRAS (median PFS 239 days versus 443 days, *P* = 0.0630). However, patients with RAS/BRAF mutations in ctDNA had a worse PFS than wild type patients (median PFS 239 days versus 443 days, *P* = 0.0173), and Cox regression analysis indicated that patients with RAS/BRAF mutation would have 3.351 (95% CI 1.172–9.576) times higher risk of disease progression that those without RAS/BRAF mutation. Our ctDNA results are in line with previous studies which also demonstrated that RAS/BRAF mutant patients are associated with reduced efficacy of first-line therapy [5]. These findings support the use of combinations of biomarkers instead of a single genotype in directing patient medical management.

Although this work provides valuable new information as well as supporting the findings of other small and large scale studies, there are limitations that should be taken into consideration when interpreting the results. One limitation of our study is that we had a relatively short duration of follow-up so we could not effectively evaluate overall survival. In addition, limited sensitivity of testing in plasma resulted in a relatively lower frequency of mutation. Finally, the small sample size of patients enrolled in this study and the number of patients lost to follow-up limited analysis of the prognostic value of ctDNA.

In conclusion, we applied next-generation sequencing instead of commonly used ARMS PCR for ctDNA gene detection in our study. NGS offered a higher throughput platform and the potential to analyze several genes simultaneously. The presence of gene mutations in ctDNA and matched primary tumor tissue was compared, the KRAS concordance between which was 81.25%. We analyzed mutations in synchronous and metachronous groups. The identical sensitivity of ctDNA in the metachronous group indicated the stable genotype status in primary tumor and blood ctDNA after disease recurrence. We also investigated the prognostic value of RAS/BRAF mutations in mCRC. Our results demonstrate that RAS/BRAF mutations as determined by plasma ctDNA detection during therapy may indicate reduced PFS. Our results contribute to a growing body of work supporting the use of ctDNA biomarkers to predict progression-free survival in patients with mCRC receiving first-line chemotherapy treatment [22]. Our findings of RAS/BRAF combination mutation value will inform others attempting similar studies and clinical trials that may lead to new tests to improve clinical outcomes. Further studies may still be needed to confirm the reliable prognostic biomarkers to improve personalized therapies and clinical management and to determine if more aggressive chemotherapies such as increased dosing or combination therapy can provide additional benefit to patients with tumor RAS/BRAF mutations.

## Figures and Tables

**Table 1:** Patient characteristics and KRAS mutation distribution.

Characteristics	*N*	Tissue KRAS_Mt	Tissue KRAS_Wt	Tissue KRAS_NA	*P* value	Plasma KRAS_Mt	Plasma KRAS_Wt	*P* value
All patients	76	24	37	15		23	53	
Age group					0.9712			0.9678
<65	46	15	22	9		14	32	
≥65	30	9	15	6		9	21	
Median (range)	62 (25–82)							
Gender					0.9121			0.6654
Male	49	16	24	9		14	35	
Female	27	8	13	6		9	18	
Primary tumor site					0.4004			0.6555
Ascending colon	7	4	2	1		2	5	
Transverse colon	4	0	2	2		1	3	
Descending colon	10	2	6	2		2	8	
Rectum, sigmoid colon	50	15	25	10		15	35	
NA	5	3	2	0		3	2	
Synchronous					0.2696			0.6005
Yes	24	10	10	4		9	15	
No	50	14	27	10		14	37	
NA	1	0	0	1		0	1	
Liver metastases					0.3253			0.7328
Yes	32	8	17	7		11	21	
No	43	16	20	7		12	31	
NA	1	0	0	1		0	1	
Perineural invasion					0.4695			0.4002
Yes	17	8	7	2		5	12	
No	43	12	23	8		11	32	
NA	16	4	7	5		7	9	
Vascular tumor thrombus					0.7652			0.3025
Yes	17	7	8	2		5	12	
No	41	12	21	8		10	31	
NA	18	5	8	5		8	10	
Number of metastatic sites					0.3196			0.3477
NA	1	0	0	1		0	1	
0	5	0	5	0		0	5	
1	30	9	14	7		8	22	
2	25	8	12	5		8	17	
>2	15	7	6	2		7	8	
Histologic grade					0.5146			0.3066
NA	15	4	9	2		5	10	
well differentiated	4	2	1	1		1	3	
moderately differentiated	48	14	25	9		12	36	
poorly differentiated	9	4	2	3		5	4	

**Table 2:** Mutation types of RAS/BRAF detected in 76 patients.

Gene	Mutation	Tissue			Plasma		
		Synchronous group	Metachronous group	Total	Synchronous group	Metachronous group	Total
KRAS	G12A, G12C, G12D, G12V, G13D, G13S	26.32% (5/19)	45.24% (19/42)	39.34% (24/61)	35.42% (17/48)	14.29% (4/28)	27.63% (21/76)
KRAS	Q61H, Q61K	5.26% (1/19)	0% (0/42)	1.64% (1/61)	0% (0/48)	10.71% (3/28)	3.95% (3/76)
KRAS	A146T	0% (0/19)	0% (0/42)	0% (0/61)	0% (0/48)	3.57% (1/28)	1.32% (1/76)
NRAS	G12V, G12D	5.88% (1/17)	0% (0/39)	1.79% (1/56)	2.08% (1/48)	0% (0/28)	1.32% (1/76)
NRAS	Q61R, Q61K	0% (0/17)	7.69% (3/39)	5.36% (3/56)	2.08% (1/48)	0% (0/28)	1.32% (1/76)
BRAF	Y472F, P403fs	0% (0/18)	0% (0/40)	0% (0/58)	2.08% (1/48)	3.57% (1/28)	2.63% (2/76)
HRAS	G12D	/	/	/	2.08% (1/48)	0% (0/28)	1.32% (1/76)
HRAS	P167fs	/	/	/	2.08% (1/48)	3.57% (1/28)	2.63% (2/76)

**Table 3:** Comparison of KRAS gene mutation in ctDNA and tumor tissue.

Plasma	Synchronous group tumor			Metachronous group tumor			Total	Sensitivity (%)	Specificity (%)	Concordance (%)
	Mt	Wt	NA	Mt	Wt	NA				
Mt	4	2	1	12	2	2	23	66.67%	90.00%	81.25%
Wt	2	14	5	6	22	4	53			
Total	6	16	6	18	24	6	76			

*Note*. Mt, mutate type; Wt, wild type; NA, not available.

## Supplementary Materials

Supplementary Materials Supplement Table 1: patients underwent a radical surgery with measurable DFS. 40 patients who underwent a radical surgery had measurable DFS, used to explore any factors that might influence DFS. Supplement Table 2: patients treatment in subsequent first-line chemotherapy with measurable PFS. The 27 patients who had been treated with subsequent first-line chemotherapy with measurable PFS were helpful in exploring factors associated with poorer PFS.

## References

[1] Ferlay J., Soerjomataram I., Dikshit R. (2014). Cancer incidence and mortality worldwide: sources, methods and major patterns in GLOBOCAN 2012. *International Journal of Cancer*.

[2] Torre L. A., Bray F., Siegel R. L., Ferlay J., Lortet-Tieulent J. (2015). Global cancer statistics, 2012. *CA: A Cancer Journal for Clinicians*.

[3] Amado R. G., Wolf M., Peeters M. (2008). Wild-type KRAS is required for panitumumab efficacy in patients with metastatic colorectal cancer. *Journal of Clinical Oncology*.

[4] Bokemeyer C., Bondarenko I., Hartmann J. T. (2011). Efficacy according to biomarker status of cetuximab plus FOLFOX-4 as first-line treatment for metastatic colorectal cancer: the OPUS study. *Annals of Oncology*.

[5] Douillard J. Y., Oliner K. S., Siena S. (2013). Panitumumab-FOLFOX4 treatment and RAS mutations in colorectal cancer. *The New England Journal of Medicine*.

[6] Van Cutsem E., Cervantes A., Adam R. (2016). ESMO consensus guidelines for the management of patients with metastatic colorectal cancer. *Annals of Oncology*.

[7] Lee D., Kim K. J., Han S. (2015). KRAS Mutation is Associated with Worse Prognosis in Stage III or High-risk Stage II Colon Cancer Patients Treated with Adjuvant FOLFOX. *Annals of Surgical Oncology*.

[8] Modest D. P., Ricard I., Heinemann V. (2016). Outcome according to KRAS-, NRAS- and BRAF-mutation as well as KRAS mutation variants: Pooled analysis of five randomized trials in metastatic colorectal cancer by the AIO colorectal cancer study group. *Annals of Oncology*.

[9] Huang C.-W., Tsai H.-L., Chen Y.-T. (2013). The prognostic values of EGFR expression and KRAS mutation in patients with synchronous or metachronous metastatic colorectal cancer. *BMC Cancer*.

[10] Mariani P., Lae M., Degeorges A., Cacheux W., Lappartient E., Margogne A. (2010). Concordant Analysis of KRAS Status in Primary Colon Carcinoma and Matched Metastasis. *Anticancer Res*.

[11] Santini D., Loupakis F., Vincenzi B. (2008). High concordance of KRAS status between primary colorectal tumors and related metastatic sites: Implications for clinical practice. *The Oncologist*.

[12] Wan R., Wang Z., Lee J. J. (2017). Comprehensive Analysis of the Discordance of EGFR Mutation Status between Tumor Tissues and Matched Circulating Tumor DNA in Advanced Non–Small Cell Lung Cancer. *Journal of Thoracic Oncology*.

[13] Overman M. J., Vauthey J-N., Aloia T. A., Conrad C., Chun Y. S., Pereira A. A. L. (2017). Circulating tumor DNA (ctDNA) utilizing a high-sensitivity panel to detect minimal residual disease post liver hepatectomy and predict disease recurrence. *Journal of Clinical Oncology*.

[14] Pietrantonio F., Petrelli F., Coinu A. (2015). Predictive role of BRAF mutations in patients with advanced colorectal cancer receiving cetuximab and panitumumab: A meta-analysis. *European Journal of Cancer*.

[15] Yaeger R., Cercek A., Chou J. F. (2014). BRAF mutation predicts for poor outcomes after metastasectomy in patients with metastatic colorectal cancer. *Cancer*.

[16] Di Nicolantonio F., Martini M., Molinari F. (2008). Wild-type BRAF is required for response to panitumumab or cetuximab in metastatic colorectal cancer. *Journal of Clinical Oncology*.

[17] Samowitz W. S., Sweeney C., Herrick J., Albertsen H., Levin T. R., Murtaugh M. A. (2005). Poor Survival Associated with the BRAF V600E Mutation in Microsatellite-Stable Colon Cancers. *Cancer Research*.

[18] Sorich M. J., Wiese M. D., Rowland A., Kichenadasse G., McKinnon R. A., Karapetis C. S. (2015). Extended RAS mutations and anti-EGFR monoclonal antibody survival benefit in metastatic colorectal cancer: a meta-analysis of randomized, controlled trials. *Annals of Oncology*.

[19] Akishima-Fukasawa Y., Ishikawa Y., Akasaka Y. (2011). Histopathological predictors of regional lymph node metastasis at the invasive front in early colorectal cancer. *Histopathology*.

[20] Courtney E. D., West N. J., Kaur C. (2009). Extramural vascular invasion is an adverse prognostic indicator of survival in patients with colorectal cancer. *Colorectal Disease*.

[21] Sugai T., Yamada N., Eizuka M. (2017). Vascular Invasion and Stromal S100A4 Expression at the Invasive Front of Colorectal Cancer are Novel Determinants and Tumor Prognostic Markers. *Journal of Cancer*.

[22] Vidal J., Muinelo L., Dalmases A. (2017). Plasma ctDNA RAS mutation analysis for the diagnosis and treatment monitoring of metastatic colorectal cancer patients. *Annals of Oncology*.

