# Supplementary material for: RAS/BRAF Circulating Tumor DNA Mutations as a Predictor of Response to First-Line Chemotherapy in Metastatic Colorectal Cancer Patients
Source: Can J Gastroenterol Hepatol. 2018 Mar 7;2018:4248971. doi: 10.1155/2018/4248971 (PMC5863340; doi:10.1155/2018/4248971)
Supplement: Supplementary Materials — Supplement Table 1: patients underwent a radical surgery with measurable DFS. 40 patients who underwent a radical surgery had measurable DFS, used to explore any factors that might influence DFS. Supplement Table 2: patients treatment in subsequent first-line chemotherapy with measurable PFS. The 27 patients who had been treated with subsequent first-line chemotherapy with measurable PFS were helpful in exploring factors associated with poorer PFS. [file 4248971.f1.pdf]

Supplement materials

Supplement table 1. Patients underwent a radical surgery with measurable DFS

Sample ID	Age	Gender	Primary tumor site	Perineural invasion	Vascular tumor thrombus	Histologic grade	T stage	N stage	NCCN stage	Tissue KRAS	Tissue Mutation	Outcome	DFS (days)
CC045	55	M	Rectum	Y	Y	moderately differentiated	T3	N2	IIIB	N	N	PD	155
CC062	68	M	Rectum	Y	Y	poorly differentiated	T3	N2a	IIIB	Y	Y	PD	247
CC063	66	M	Rectum	Y	Y	moderately differentiated	T3	N1b	IIIB	N	N	PD	51
CC064	69	M	Rectum	Y	N	moderately differentiated	T3	N1b	IIIB	Y	Y	PD	92
CC065	77	M	Rectum	N	N	moderately differentiated	T3	N1a	IIIB	N	N	PD	1837
CC070	79	F	Rectum	Y	Y	moderately differentiated	T3	N1	IIIB	Y	Y	PD	60

CC071	65	F	Descending colon	N	N	NA	T2	N2b	IIIA	N	N	PD	106
CC075	53	F	Rectum	N	N	moderately differentiated	TX	N1a	NA	N	N	PD	519
CC076	66	M	Descending colon	N	N	moderately differentiated	T3	N1b	IIIB	N	N	PD	417
CC077	70	M	colon	Y	N	NA	T3	N0	IIA	N	N	PD	1100
CC079	43	M	Descending colon	N	N	moderately differentiated	T3	N1b	IIIB	N	N	SD	979
CC081	71	F	Descending colon	N	N	moderately differentiated	T3	N0	IIA	N	N	PD	322
CC084	63	M	colon	N	N	moderately differentiated	T3	N2	IIIB	Y	Y	PD	365
CC086	68	M	Rectum	Y	Y	moderately differentiated	T3	N1	IIIB	Y	Y	PD	648
CC089	52	M	Rectum	NA	NA	poorly differentiated	T3	N2	IIIB	N	N	PD	637

CC091	67	M	Rectum	N	N	moderately differentiated	T2	N2	IIIB	Y	Y	PD	203
CC102	76	M	Sigmoid colon	NA	NA	moderately differentiated	T2	N0	I	N	Y	PD	1360
CC106	61	M	Rectum	Y	N	moderately differentiated	T3	N2a	IIIB	N	N	SD	670
CC111	50	F	Sigmoid colon	Y	NA	moderately differentiated	T4	N1	IIIB	Y	Y	PD	303
SOP22	74	M	Ascendi ng colon	N	N	moderately differentiated	T3	N2	IIIB	Y	Y	PD	578
SOP25	52	M	Rectum	N	Y	moderately differentiated	T3	N1	IIIB	N	N	PD	350
SOP27	55	M	Rectum	N	N	moderately differentiated	T3	N1	IIIB	N	N	PD	655
SOP29	60	M	Sigmoid colon	N	N	moderately differentiated	T3	N0	IIA	N	N	PD	692
SOP30	59	F	Rectum	N	N	NA	T2	N1	IIIA	N	N	SD	2841

SOP41	61	M	Rectum	N	Y	moderately differentiated	T3	N1b	IIIB	N	N	PD	593
SOP44	57	M	Rectum	N	N	moderately differentiated	T4	N1	IIIB	N	N	PD	105
SOP48	60	M	Rectum	N	N	NA	T3	N1	IIIB	N	N	PD	65
SOP49	74	F	Sigmoid colon	N	N	moderately differentiated	T3	N1	IIIB	N	N	PD	729
SOP5	52	M	Rectum	NA	NA	NA	T3	N0	IIA	N	N	PD	182
SOP50	55	M	Ascendi ng colon	N	N	moderately differentiated	T3	N2	IIIB	N	N	PD	73
SOP51	71	F	Rectum	N	Y	moderately differentiated	T3	N0	IIA	Y	Y	PD	932
SOP56	52	F	Rectum	N	N	well differentiated	T4	N1	IIIB	Y	Y	PD	1155
SOP61	52	M	Ascendi ng colon	N	N	moderately differentiated	T3	N0	IIA	Y	Y	PD	700
SOP62	76	M	Sigmoid colon	N	Y	NA	T4	N2	IIIC	N	N	PD	247

SOP68	43	F	Descending colon	N	N	moderately differentiated	TX	N1b	NA	Y	Y	PD	313
SOP70	56	M	Rectum	Y	Y	moderately differentiated	T3	N2	IIIB	Y	Y	PD	271
SOP74	46	M	Ascending colon	Y	N	NA	T4	N1	IIIB	Y	Y	PD	310
SOP77	51	M	Sigmoid colon	N	N	moderately differentiated	T3	N2	IIIB	N	N	SD	184
SOP80	62	F	Sigmoid colon	N	N	poorly differentiated	TX	N2b	NA	Y	Y	PD	854
SOP89	61	M	Descending colon	Y	N	moderately differentiated	T4	N1a	IIIB	N	N	PD	590

Supplement table 2. Patients treatment in subsequent first line chemotherapy with measurable PFS

Sample ID	Age	Sex	Primary Tumor Site	Perineural Invasion	Vascular tumor Thrombus	Histologic Grade	Number of metastatic site	T	N	NCCN stage	Plasma KRAS	Plasma Mutation	Outcome	PFS (days)
CC052	59	F	Rectum	N	N	well differentiated	2	T2	NX	IV	Y	Y	PD	141
CC062	68	M	Rectum	Y	Y	poorly differentiated	1	T3	N2a	IV	Y	Y	SD	147
CC064	69	M	Rectum	Y	N	moderately differentiated	2	T3	N1b	IV	Y	Y	PD	204
CC065	77	M	Rectum	N	N	moderately differentiated	1	T3	N1a	IV	N	N	SD	126
CC068	62	M	Rectum	N	N	NA	2	T2	N1	IV	N	N	PD	302
CC070	79	F	Rectum	Y	Y	moderately differentiated	1	T3	N1	IV	N	N	PD	50

CC071	65	F	Descending colon	N	N	NA	1	T2	N2b	IV	N	N	PD	357
CC074	80	M	Transverse colon	N	N	moderately differentiated	1	T3	N1b	IV	N	N	SD	272
CC077	70	M	colon	Y	N	NA	3	T3	N0	IV	N	Y	PD	49
CC081	71	F	Descending colon	N	N	moderately differentiated	2	T3	N0	IV	N	N	SD	1308
CC084	63	M	colon	N	N	moderately differentiated	1	T3	N2	IV	Y	Y	PD	44
CC086	68	M	Rectum	Y	Y	moderately differentiated	1	T3	N1	IV	Y	Y	SD	598
CC091	67	M	Rectum	N	N	moderately differentiated	2	T2	N2	IV	Y	Y	PD	350
CC102	76	M	Sigmoid colon	NA	NA	moderately differentiated	2	T2	N0	IV	N	N	PD	443

CC108	57	M	Rectum	Y	Y	moderately differentiated	2	T4	N2	IV	N	N	SD	609
SOP22	74	M	Ascending colon	N	N	moderately differentiated	3	T3	N2	IV	N	N	SD	487
SOP25	52	M	Rectum	N	Y	moderately differentiated	2	T3	N1	IV	N	N	PD	281
SOP27	55	M	Rectum	N	N	moderately differentiated	2	T3	N1	IV	N	N	SD	518
SOP29	60	M	Sigmoid colon	N	N	moderately differentiated	2	T3	N0	IV	N	N	PD	111
SOP44	57	M	Rectum	N	N	moderately differentiated	3	T4	N1	IV	N	N	PD	264
SOP49	74	F	Sigmoid colon	N	N	moderately differentiated	3	T3	N1	IV	N	N	SD	270
SOP51	71	F	Rectum	N	Y	moderately differentiated	1	T3	N0	IV	Y	Y	PD	239

SOP56	52	F	Rectum	N	N	well differentiated	1	T4	N1	IV	N	N	SD	451
SOP61	52	M	Ascending colon	N	N	moderately differentiated	1	T3	N0	IV	Y	Y	PD	259
SOP63	82	F	Transverse colon	N	N	moderately differentiated	3	T4	NX	IV	Y	Y	PD	44
SOP70	56	M	Rectum	Y	Y	moderately differentiated	2	T3	N2	IV	Y	Y	SD	158
SOP89	61	M	Descending colon	Y	N	moderately differentiated	1	T4	N1a	IV	N	N	SD	59
